# Empirically supported interventions in psychology: contributions of Research Domain Criteria

**DOI:** 10.1186/s41155-019-0128-1

**Published:** 2019-07-22

**Authors:** Rita Pasion, Eva C. Martins, Fernando Barbosa

**Affiliations:** 1 Laboratory of Neuropsychophysiology, Faculty of Psychology and Educational Sciences, University of Porto, Rua Alfredo Allen, 535, 4200-135 Porto, Portugal; 2 Department of Social and Behavioural Sciences (CPUP), University of Porto, Porto, Portugal; 3 Maia University Institute (ISMAI), Maia, Portugal

**Keywords:** Research Domain Criteria (RDoC), Empirically based psychological interventions, Anxiety, Depression

## Abstract

Empirically supported interventions in psychological disorders should provide (1) evidence supporting the underlying psychological mechanisms of psychopathology to target in the intervention and (2) evidence supporting the efficacy of the intervention. However, research has been dedicated in a greater extent to efficacy than to the acquisition of empirical support for the theoretical basis of therapies. Research Domain Criteria (RDoC) emerges as a new framework to provide empirically based theories about psychological mechanisms that may be targeted in intervention and tested for its efficacy. The current review aims to demonstrate the possible applications of RDoC to design empirically supported interventions for psychological disorders. Two RDoC-inspired interventions are reviewed, and the RDoC framework is broadly explored in terms of its contributions and limitations. From preliminary evidence, RDoC offers many avenues for improving evidence-based interventions in psychology, but some limitations must be anticipated to increase the RDoC applicability to naturalistic settings.

## Introduction

The search for empirically supported interventions of psychological disorders has marked a paradigm shift in applied psychology. This movement followed changes in other areas like medicine and resulted from the need to provide clinicians with clear directives regarding the psychological treatments that were effective and the ones that were not, or even worse, were harmful. Hence, APA’s Presidential Task Force on Evidence-Based Practice in Psychology stressed the importance of incorporating empirically supported treatments with clinical expertise in the context of patient characteristics, culture, and preferences (Levant, 2005). Although APA accepts many forms of research evidence (Levant, 2005), most of the studies are centered on whether the treatments work and in what conditions (Roberts, Blossom, Evans, Amaro, & Kanine, 2017).

Nevertheless, the focus on efficacy in evidence-based interventions in psychological disorders (e.g., if the intervention is associated with symptom reduction) has not been paralleled with the acquisition of empirical support for the theoretical basis of these therapies (Emmelkamp et al., 2014). Evidence-based interventions should have an empirical background supporting the psychological mechanisms of psychopathology targeted for intervention and not only present the efficacy of the therapeutic package (David & Montgomery, 2011).

The smaller emphasis on background evidence may be explained by the state of the art in psychology and the yet incipient comprehension of mental disorders. For one, there is a wide variety of models regarding mental disorders and, subsequently, longstanding debates in the field. For the other, psychological and neurodevelopmental research lacks integration into a comprehensive conceptual framework of mechanisms in mental disorders, which constitutes a roadblock for designing empirically sound interventions in psychology (Emmelkamp et al., 2014). In this sense, the Research Domain Criteria (RDoC) movement emerges as a framework for dealing with these limitations.

The goal of this review is to explore the RDoC and discuss its relevance for designing empirically supported interventions in psychological disorders. We will start by introducing the RDoC framework and by examining its practical applications in the clinical setting. Then, two psychological interventions—training for awareness resilience and action (TARA) and Engage—that claim to be inspired by the RDoC framework in terms of its empirically based foundations and preliminary evidence on efficacy will be used to illustrate some of the possible applications of RDoC in psychological clinical settings. Then, the final section of this article aims to provide a critical analysis of RDoC applicability to real-life psychological interventions, from the observed advances and obstacles exposed by TARA and Engage at conceptual, methodological, and efficacy measurement levels.

## The RDoC framework

RDoC was launched by the National Institute of Mental Health in 2009 as a new approach towards an empirically based model of psychopathology (Cuthbert & Insel, 2013; Insel et al., 2010) that is bound beyond experts’ opinion (e.g., DSM) (Appelbaum, 2017; Clark, Cuthbert, Lewis-Fernández, Narrow, & Reed, 2017). Thus, the RDoC framework was envisioned to provide a comprehensive understanding of psychopathology (and its mechanisms) based on empirical findings, ultimately allowing for the development of reliable diagnosis, as well as evidence-based pharmacological and psychological interventions (Morris, Rumsey, & Cuthbert, 2014). Although it may be argued that decision-making may still be biased and influenced by external factors, the decisions of the RDoC Task Forces are necessarily supported by the available research.

These efforts gave rise to a matrix that is the heart of RDoC. The RDoC matrix depicts biopsychological processes (represented by constructs within higher-order functional domains) and their expression (represented by units of analysis) to assess phenotypic expressions of psychopathology alongside with their interconnected mechanisms (for an overview of the RDoC matrix see https://www.nimh.nih.gov/research-priorities/rdoc/constructs/rdoc-matrix.shtml). At this point, it is important to note that the current matrix is a work in progress and not a final/static description of the human biopsychological aspects (Clark et al., 2017). As the framework provided by the RDoC matrix is dependent on the emergent empirical findings, the matrix had several revisions to date. Until recently, there were five functional domains in the RDoC matrix—positive valence systems, negative valence systems, cognitive control systems, arousal and regulatory systems, and social processes systems—but at the beginning of 2019 the Sensory Systems domain was added to encourage research focused on the role of motor systems disruptions across mental disorders (for a full description of the domains and constructs see https://www.nimh.nih.gov/research-priorities/rdoc/constructs/index.shtml).

Each of these six domains is divided into constructs capturing more specific dimensions (e.g., loss is a construct of the negative valence system domain), which may be used to investigate each mechanism of biopsychological functioning. For this purpose, the constructs are divided into units of analysis. These units of analysis consist of different methods—from molecules and neuronal-circuit activity to behavior and self-reports—to measure the expression of the underlying constructs. The inclusion of distinct unit of analysis represents an effort to encourage translational research and makes the RDoC matrix a multidisciplinary entity, allowing for multi-level analysis (Cuthbert & Insel, 2013) and for aggregating research streaming from biology and neuroscience to psychology (Kozak & Cuthbert, 2016). Currently, an investigation from these different areas is rarely integrated into a causal model of psychopathology. Most basic and applied research is disconnected leading to major breakthroughs in the development of better interventions (Morris et al., 2014).

Importantly, the constructs are defined as having at one end of the continuum its normal range and at the other end of the continuum its psychopathological forms, as well as having a transdiagnostic identity that cuts across mental disorders as they are defined today (e.g., DSM) (Kozak & Cuthbert, 2016). In this sense, RDoC rethinks psychopathology from the perspective of domains of function and basic mechanisms and does not consider psychological disorders as currently operationalized in a cluster of symptoms. This new approach may promote a better understanding of psychopathology that will eventually take us to better and earlier diagnosis and improved and tailored interventions (Clark et al., 2017).

In sum, by providing a “map” of constructs that can be tested simultaneously along many levels of analysis, RDoC allows to obtain evidence-based validity for mental disorders and its mechanisms, theories about development and developmental psychopathology, and finally, for evidence-based interventions. Specifically, the last topic will be addressed in more detail in the next sections.

## RDoC empirically based interventions

As explained in the previous section, the RDoC-inspired research gave way to the construction of the RDoC matrix, a framework for studying relevant biopsychological constructs and its behavioral manifestations. The main goal was to integrate knowledge that was being acquired from different fields of study (e.g., psychology, neuroscience, biology) so that better interventions would be available for the treatment of mental disorders (Cuthbert & Insel, 2013; Morris et al., 2014).

Of special relevance for the topic of this article, RDoC is a new path towards providing validity evidence for the theory about psychological mechanisms of psychopathology and towards improving the way how efficacy in intervention is measured, in terms of change in the targeted mechanisms featured in the intervention. The absence of the first has been stressed as one of the major limitations in the evaluative framework of evidence-based interventions in psychology (David & Montgomery, 2011; Emmelkamp et al., 2014).

However, an initial challenge to the design of an RDoC informed intervention emerges from the assumption that RDoC does not recognize diagnosis a priori, for example, DSM categories (Cuthbert & Kozak, 2013). RDoC informed researchers of psychopathology are currently working with the RDoC matrix to push the state of art towards the stage 0 (basic science research prior to intervention) (Shoham et al., 2014), by including large datasets and by collecting multi-level data across multi-domains (e.g., Clementz et al., 2016; Drysdale et al., 2016; Van Dam et al., 2017). Ultimately, the accumulated scientific evidence will allow to isolate empirically derived clusters (biotypes) that may inform researchers how to design interventions that go beyond the current (heterogeneous) categorical diagnosis and that are focused on more specific-unique profiles of psychopathology (Clark et al., 2017; Clementz et al., 2016). As such, it will be possible in the long term to refine interventions for groups that are heterogeneous in nature and show divergent responses to the available treatments.

Nonetheless, the process of redefining psychopathology from RDoC-inspired research is still at the beginning and is highly demanding. For now, one possible venue is to test whether the RDoC constructs described in the matrix are mechanisms of change and promote efficacy in psychological interventions. TARA and Engage—the two interventions that will be analyzed in the current article—had started to use the empirical RDoC constructs to conceive new dimensional interventions in psychology.

At this point, it is important to systematize some practical aspects that must be taken into account when operationalizing the RDoC framework in the psychological clinical setting.

### RDoC-based practical applications to interventions

Considering the RDoC postulates, there are some aspects that may be expected when designing and testing RDoC evidence-based interventions. As described below, these aspects may provide some guidance to clinicians aiming to design RDoC-inspired interventions.

First, it is important to isolate the RDoC constructs of interest with functional relevance to the psychopathological phenomena. The RDoC matrix was built on the assumption that neurobiological knowledge has reached the point of providing biologically meaningful targets, thus guiding the development of empirically informed interventions (Alexopoulos & Arean, 2014). Therefore, this framework informs clinicians about valid theories of psychopathology, allowing to better decide on which RDoC constructs should be used as targets in therapies. From the current standpoint, it is possible to roughly arrange the existing diagnoses into components of the RDoC matrix to select the constructs for intervention with high functional relevance to a disorder or a cluster of interrelated disorders (McKay & Tolin, 2017). For example, anxiety disorders are closely associated with the RDoC negative valence systems domain along the constructs of acute, sustained, and potential threat constructs (McKay & Tolin, 2017), and also with some constructs of the cognitive control systems domain (e.g., performance-monitoring subconstruct) (Pasion, Paiva, Fernandes, Almeida, & Barbosa, 2018).

Second, and from the set of selected constructs, it is important to develop a hierarchy of priority to intervention (Alexopoulos & Arean, 2014). This is one of the ways how RDoC promotes tailored interventions at the individual level (Clark et al., 2017). Alexopoulos and Arean (2014) recommended personalizing the intervention by selecting the appropriate psychological, behavioral, and ecosystem modification strategies to target abnormal domains of functioning and the respective constructs while considering additional domains and constructs in patients unresponsive to treatment. As such, the individual variability is taken into account independently of the previous diagnosis. Two individuals with anxiety may show distinct RDoC-related dysfunctions, and subsequently, the intervention must be necessarily distinct (Hershenberg & Goldfried, 2015).

Third, and to apply the hierarchical analysis of constructs to intervention, it is recommended to disentangle risk factors from mediation and moderation effects (Hershenberg & Goldfried, 2015). Mediator (how a treatment works) and moderator mechanisms (for whom treatment works) of effective treatment need to be accommodated in the intervention design (Hershenberg & Goldfried, 2015; McKay & Tolin, 2017). To the extent that various treatments operate via different mechanisms, this may facilitate the development of innovative and synergistic treatments, maximizing active therapeutic components and minimizing inactive or antagonistic ingredients, while anticipating which subpopulations will respond to a given intervention.

Forth, some methodological issues should be considered. The conceptual distance from categorical clinical diagnosis argues for the usage of large sample sizes in intervention research and for the inclusion of comorbid conditions that share causal (basic) mechanisms (Clementz et al., 2016; Drysdale et al., 2016; Van Dam et al., 2017). For example, threat-related and cognitive control constructs are not only relevant for anxiety disorders, but also for obsessive-compulsive and post-stress traumatic disorders (McKay & Tolin, 2017). In this sense, another common RDoC recommendation is to avoid extensive exclusion criteria that may limit the intended cohort approximation of a representative sample and a misrepresentation of the psychopathological spectrum (Van Dam et al., 2017). The inclusion of less severe expressions of psychopathology or even healthy relatives may also be motivated by the continuum analysis of the normal-abnormal spectrum.

Finally, and consistently with empirically based ambitions, it is mandatory to examine whether the efficacy of the new intervention is explained by alterations in the purported mechanisms of change. Since the units of analysis enable to better isolate the indicators of change, this may be demonstrated by collecting multi-level data (i.e., self-report, molecular, cellular, circuit, physiological, and behavioral data) across the selected multi-domains and constructs for intervention. As such, it is important to include the proposed RDoC units of analysis in the study design—along with other measures of interest to researchers. The RDoC matrix intends to be universal and, thus, a consistent application of the same constructs and units of analysis may foster the collection of uniformized data across distinct research groups. RDoC further calls for replication longitudinal analysis to prove the generalization and stability of the findings.

From this demonstration of how RDoC may work in clinical settings and intervention research, the next sections will illustrate how TARA and Engage were conducted to put into practice some of the RDoC postulates.

## TARA

TARA is a group intervention program for adolescents with depression (14–18-year-olds), but also includes participants with anxiety symptoms. It comprises 12 weekly sessions of 90 min, in groups up to 12 participants. The program is composed of four modules (apiece lasting 3 sessions): Module 1, calming down and creating a sense of safety; Module 2, attending and caring about our inner experience; Module 3, recognizing, regulating and communicating emotions; and finally, Module 4, core values, goal setting, and committed action (Blom et al., 2014, 2017).

The TARA program is one of the first published attempts to develop a psychological intervention that is translational in nature. Put differently, TARA authors were inspired by neuroscience-based research findings on RDoC mechanisms in depression and targeted them to intervention (Blom et al., 2017). On first glance, TARA seems to be just another mindfulness-based intervention (Blom et al., 2017). Undeniably, the intervention technics used in TARA have long been part of the repertoire of mindfulness, action and commitment therapy, and cognitive-based interventions. However, if we trace back to how these technics were developed and integrated into the aforementioned therapies, we realize that they lacked, in their inception, neuroscience evidence supporting them. On the contrary, empirical-based therapies in RDoC aim to integrate neuroscientific findings to support the goals of the intervention (psychological mechanisms) and the intervention technics tailored to achieve these goals. This will be explored in the next section, when we map the empirical-based RDoC constructs and neurodevelopmental research in depression, which were the basis to provide some guidelines to TARA’s intervention foci. TARA development was influenced by RDoC dimensional/transdiagnostic mindset, by including participants with depressive and/or anxiety symptoms due to the high comorbidity between these disorders and the common underlying mechanisms (e.g., genetic risk) evidenced by neuroscientific findings (Blom et al., 2017).

The TARA program targets many RDoC constructs and domains. However, we will focus on the ones reviewed in the last publication (Blom et al., 2017): *sustained threat*, *loss*, *social processes*, and *reward learning*.

### Negative valence systems: sustained threat construct

*Sustained threat* is one of the constructs included in the domain of *negative valence systems,* associated with the dysregulation of amygdala reactivity and its brain circuits (*units of analysis*). Sustained threat is defined as “An aversive emotional state caused by prolonged (i.e., weeks to months) exposure to internal and/or external condition(s), state(s), or stimuli that are adaptive to escape or avoid. The exposure may be actual or anticipated; the changes in affect, cognition, physiology, and behavior caused by sustained threat persist in the absence of the threat and can be differentiated from those changes evoked by acute threat” (RDoC, 2019).

The TARA program focused on the construct of sustained threat as one of the processes involved in adolescent depression because several neuroimaging studies have demonstrated that depressed adolescents show increased hyperactivation of the amygdala and alterations in its connectivity (Blom et al., 2014, 2017). This has been associated with persistent mood and vegetative symptoms experienced by adolescents with depression (e.g., dysphoria, lassitude) potentially via impaired processing of memories and visceral signals (Cullen et al., 2014). Hence, the first intervention target of TARA was to increase vagal and sensory afference through breathing practices and synchronized movement (Module 1), aiming at diminishing amygdala hyperactivation through a bottom-up approach. Supporting this option, recent research using neuronal recordings from rats revealed that the activity in the amygdala was altered following vagal nerve stimulation (Alexander et al., 2017). The authors of the program expected that these intervention strategies would result in decreased feelings of stress, as well as physical symptoms of depression and anxiety, allowing for improved emotional self-regulatory abilities and sleep (Blom et al., 2017).

### Negative valence systems: loss construct

*Loss* is also included in the domain of negative valence systems and is associated with brain circuits (units of analysis) like the default mode network and the amygdala. Loss is defined as “A state of deprivation of a motivationally significant con-specific, object, or situation. Loss may be social or non-social and may include permanent or sustained loss of shelter, behavioral control, status, loved ones, or relationships. The response to loss may be episodic (e.g., grief) or sustained” (RDoC, 2019).

The construct of loss was featured in the TARA program as several neuroimaging studies showed alterations in the DMN (default mode network) in depressed adolescents (Blom et al., 2014, 2017), as well as in their amygdala restating state functional connectivity (Clark et al., 2018). At the same time, the heightened dominance of the DMN has been associated with higher levels of maladaptive, depressive rumination, and lower levels of adaptive, reflective rumination (Hamilton et al., 2011). Also, there is enough evidence for a relation between disrupted amygdala-prefrontal connectivity and stress-related rumination in depressed adolescents (Fowler, Miernicki, Rudolph, & Telzer, 2017). This self-criticism and broader negative expectations about the self, future, and others are corner symptoms of depression (Beck & Bredemeier, 2016) supporting the role of the construct of loss as one of its basic mechanisms. Therefore, the second intervention target was to help participants shift neuronal activation away from the DMN by firstly noticing negative self-referential thoughts and then focusing on the present sensory and interoceptive input, including practices of identifying, labeling, and expressing emotional processes without judgment (Module 2). Indeed, mindfulness training in adults has been associated with deactivation of the DMN (Berkovich-Ohana, Glicksohn, & Goldstein, 2012) and altering of resting-state functional connectivity of the amygdala (Taren et al., 2015). Based on these results, the mindful-based practices seem good intervention strategies for decreasing rumination and generalized anxiety, which were the expected outcomes of Module 2 (Blom et al., 2017).

### Social processes: social communication, perception, and understanding of self and others constructs

The *systems for social processes* is the functional domain that “mediate[s] responses to interpersonal settings of various types, including perception and interpretation of others’ actions” (RDoC, 2019). Many of the constructs of this domain were featured in the TARA intervention, for instance, social communication, perception, and understanding of self and others (Blom et al., 2014). They are the focus of Module 3. Authors state that because there are many brain circuits involved in these constructs, they did not emphasize one in particular (Blom et al., 2014).

The *social processes systems* is a domain of interest, as it has been shown that interpersonal problems (e.g., social conflict and social rejection), whether in the family realm or not, are relevant distal factors or proximal precipitants of depression (Beck & Bredemeier, 2016; Blom et al., 2017). Therefore, the third target of TARA program was to develop in the participants a set of emotion regulation skills for dealing with social interactions. These skills involved recognizing emotional triggers in oneself and others, empathetic listening, effective communication, and compassionate responses to oneself and others (Blom et al., 2017). Prior research has demonstrated that mindfulness intervention, which explicitly coaches self-compassion and mindful exposure to social situations, is associated with better social adjustment and lower social anxiety symptoms (Koszycki et al., 2016). The authors of the program used diminished levels of social anxiety as evidence that the intervention would have resulted in an improvement in the abovementioned social processes (Blom et al., 2017).

### Positive valence systems: reward learning construct

Is one of the constructs included in the domain of *positive valence systems,* associated with brain circuits (units of analysis) that include the ventral and dorsal striatum. *Reward learning* is defined as “A process by which organisms acquire information about stimuli, actions, and contexts that predict positive outcomes, and by which behavior is modified when a novel reward occurs or outcomes are better than expected. Reward learning is a type of reinforcement learning, and similar processes may be involved in learning related to negative reinforcement” (RDoC, 2019).

Reward learning was selected as one of the constructs to be included in TARA because depression has been associated with the disrupted balance of cortico-striatal circuit function in adolescents (Blom et al., 2014, 2017; Kerestes et al., 2015). These circuitries have been implicated in emotional processing, reward learning, and cognitive control, which in turn have been highlighted as some of the mechanisms in depression. Indeed, impaired reward learning may hinder changing negative self-referencing thinking based on new positive experiences (Marchand, 2012) and contribute to low mood, anhedonia, and psychomotor retardation (Price & Drevets, 2012). Hence, the fourth intervention target of TARA was to increase behavioral activation guided by intrinsic reward. These strategies were inspired by acceptance and commitment therapy, which share many of the mindfulness and cognitive restructuration strategies (Module 4). Here, participants had to develop a personal strategy for living their life in coherence with their own values, had to challenge patterns of experiential avoidance, and practiced top-down cognitive control of affective responses (Blom et al., 2014). The expected behavioral outcomes of this module were decreased experiential avoidance, increased committed action, and improved behavior during emotional arousing experiences (Blom et al., 2017).

### Research evidence supporting TARA

There is one pilot study published with TARA. A single-arm trial was conducted with 26 adolescents (14–18-year-olds; 19 females). Participants met inclusion criteria when they had clinically significant depressive and/or anxious symptomatology. There were three assessment time points: before the groups started; after the groups had finished; and 3 months after TARA ended. Outcomes were reduction of adolescent depression (anhedonia/negative affect, dysphoric mood, negative self-evaluation, somatic complaints); anxiety (physical symptoms, harm/avoidance, social anxiety, separation/panic), insomnia severity, and experiential avoidance; and increase in mindfulness.

There is preliminary evidence that TARA may be a feasible and efficacious intervention program. Overall, TARA participation was associated with less symptoms of depression and generalized and social anxiety, even 3 months later (Blom et al., 2017).

By analyzing changes in the RDoC domains/constructs, using the self-reports as units of analysis, some validity evidence is obtained not only for the hypothesized mechanisms in depression but also for the efficacy of TARA modules/techniques in changing such processes. Indeed, the pilot study’s results point to some improvements in the RDoC domains/constructs. From baseline to 3 months after the end of the intervention, participants reported (Blom et al., 2017) fewer physical symptoms of anxiety/depression, insomnia, and improved self-regulatory skills (Module 1—construct of sustained threat), decrease in rumination (Module 2—construct of loss; decrease in social anxiety symptoms (Module 3—domain of social processes, and reduction in anhedonia and experiential avoidance (Module 4—construct of reward learning).

## Engage

Engage is a structured, stepped approach therapy for late-life depression with nine sessions of 40–45 min each (Alexopoulos et al., 2015, 2017; Alexopoulos & Arean, 2014). The Engage key RDoC constructs for intervention were defined after consider the input provided by professionals in the field. The authors were guided by the principle of simplification when defining the main strategies for intervention, based on the assumption that simplifying the language and strategies when disseminating RDoC-inspired interventions may turn them more accessible to professionals. Moreover, Engage is based on the behavioral activation therapy (Alexopoulos & Arean, 2014), as it is aligned with the broad perspective that depression is associated with a loss of reinforcement from the environment leading to inadequate experience of pleasure or meaning from rewards (Ferster, 1973). Since individuals lose interest in activities, leading to melancholic mood, anhedonia, and psychomotor retardation, the intervention entails the scheduling and facilitation of meaningful, rewarding activities. “Reward exposure” is, therefore, the main focus of Engage and the therapeutic vehicle is the RDoC positive valence system (Alexopoulos et al., 2015; Alexopoulos & Arean, 2014).

In line with empirically based ambitions, the authors of Engage relied on the RDoC workshop proceedings and related findings to select intervention targets, assuming that the RDoC framework reflects an empirical organization of the accumulated knowledge. The end result was a simplified intervention targeting domains of function and constructs with high relevance in late-life depression. It is worth noticing that reward learning, a construct of this system, was also a target for intervention in Blom et al. (2017), but the reviewed evidence and the intervention design account for the specificities of the targeted group (adolescent vs. elderly). Personalized therapies are, in fact, an RDoC assumption. The domains and constructs are not designed to be independent, but rather promote comprehensive and tailored interventions (Clark et al., 2017). In other words, it is not intended to isolate specific constructs to intervention, but rather consider multiple RDoC constructs of interest while defining a unique profile of intervention (Alexopoulos & Arean, 2014; Geraldo, Azeredo, Pasion, Dores, & Barbosa, 2018). Despite the focus on positive valence system—the core system to explain depression in later-life—the Engage program also includes additional constructs that may moderate the efficacy in intervention, namely loss, arousal, and cognitive control (Alexopoulos et al., 2015, 2016, 2017; Alexopoulos & Arean, 2014).

### Positive valence systems: approach motivation and reward learning constructs

The RDoC positive valence system is proposed by the authors (Alexopoulos et al., 2015, 2016, 2017; Alexopoulos & Arean, 2014) as a central mechanism to late-life depression, since the alterations on approach motivation and reward learning constructs seem to hinder rewarding activities, strengthening depressive symptoms and beliefs of lack of meaning of life.

Approach motivation is a “multi-faceted construct involving mechanisms/processes that regulate the direction and maintenance of approach behavior influenced by pre-existing tendencies, learning, memory, stimulus characteristics, and deprivation states” (RDoC, 2018). *Reward valuation* and *action selection/preference-based decision-making* are subconstructs of approach motivation.

It is important to note, however, that in the last version of the RDoC matrix launched at the beginning of 2019, the positive valence systems had been reorganized. *Approach motivation* has been removed from the matrix and Reward Valuation is now a construct itself (rather than a subconstruct of approach motivation). Reward Valuation is defined as the set of “Processes by which the probability and benefits of a prospective outcome are computed by reference to external information, social context (e.g., group input), and/or prior experience. This computation [that may involve the assignment of incentive salience to stimuli] is influenced by preexisting biases, learning, memory, stimulus characteristics, and deprivation states” (RDoC, 2019). Importantly, Reward Valuation includes Delay as a subconstruct that is enrolled in action selection and decision-making as it represents “processes by which the value of a reinforcer is computed as a function of its magnitude and the time interval prior to its expected delivery” (RDoC, 2019).

Despite the changes to the matrix in which Engage was based on, the background remains consistent and valuable. As the authors had stated, abnormalities in Reward Valuation are detected in time inconsistency and intertemporal action for gains and losses in delay discounting tasks (Alexopoulos et al., 2015, 2016, 2017; Alexopoulos & Arean, 2014).

In what regards reward learning, a construct previously explored in TARA, low functional activation of the nucleus accumbens, striatum, and the caudate during the processing of positive-rewarding stimuli in monetary paradigms supports deficits in the acquisition of information that predict positive outcomes (Alexopoulos et al., 2015, 2016, 2017; Alexopoulos & Arean, 2014),.

Thus, considering the dysfunction in constructs of the positive valence system, the Engage program uses reward exposure to reignite this system in terms of reinforcing the engagement in social and physical rewarding activities that have been abandoned by depressed patients. The authors acknowledge that rewarding activities have been a central component of most psychotherapies, where therapists guide patients to develop a list of meaningful activities, such as social engagement, physical exercise, and intellectual exchange, and noticed that depressed elderlies are responsive to these activities.

During the first three sessions, and in a context of a supportive relationship, the focus is on a direct facilitation of rewarding activities using a simplified problem-solving approach: (a) select the most feasible and rewarding goal of the activities included in the participants’ list, (b) develop a list of ideas on what to do in order to meet this goal, (c) choose one of these ideas, and (d) make an “action plan” with specific steps to address obstacles. These strategies allow to operationalize approach motivation.

As a therapeutic tool, these strategies also constitute a behavioral probe that allows to identify patients who fail to engage in rewarding activities. At the end of the third session, therapists conduct a structured assessment to determine whether patients (a) learned how to form action plans, (b) engaged in the planned activities, and (c) showed improvement in depression scores. If all conditions are met, patients continue with reward exposure to promote reward learning, moving from easy to more complex activities. If not, therapists identify barriers and strategies to mitigate them, so that “reward exposure” can progress unhindered. Barriers such as negativity bias, apathy, and emotional dysregulation may be behavioral expressions of neurobiological system dysfunctions frequently occurring in late-life depression (Alexopoulos & Arean, 2014). Each of these barriers are related to impairment in RDoC domains of functioning—respectively, loss, arousal, and cognitive control—and are recurrent targets for learning-based therapies. Cognitive-behavioral and ecosystem modification strategies that literature has found efficacious are then used, as detailed below. A similar assessment is made at the end of session 6, and for those still experiencing difficulties, therapists assess whether other barriers exist and add strategies to counteract them.

As a general outcome, it is expected an increase in behavioral activation and engagement in rewarding activities associated with a reduction in depressive symptoms.

### Negative valence systems: loss construct

The negativity bias, that is the tendency to redirect the attention towards negative rather than positive information, is a vulnerability factor for depression thought to reflect the behavioral expression of the RDoC Loss construct described in the negative valence systems (Alexopoulos & Arean, 2014) and previously described TARA.

Correlates of negativity bias include neural excitation to signals of potential danger, heightened startle amplitude, higher heart rate, higher functional activation of the amygdala, anterior cingulate cortex, and ventral and dorsomedial prefrontal cortex to conscious and nonconscious fear probes; moreover, the 5-HTTLPR serotonin transporter seems to explain the heightened sensitivity to negative stimuli (Alexopoulos et al., 2015; Alexopoulos & Arean, 2014).

Several simplified cognitive-behavioral strategies are used to mitigate the negativity bias, by redirecting the attention to neutral or positive aspects of the reward exposure, namely play devil’s advocate for thoughts interfering with engagement, weigh the evidence to motivate to pursue activities, practice the positivity bias, write alternative positive explanations to negative thoughts, and learn how positive people respond to the same negative situations (Alexopoulos et al., 2016, 2017; Alexopoulos & Arean, 2014).

### Arousal and regulatory system: arousal construct

Apathy, the lack of motivation not attributable to a diminished level of consciousness, cognitive impairment, or emotional distress seems to constitute the behavioral expression of a dysfunction in the arousal and regulatory system, particularly in Arousal (Alexopoulos & Arean, 2014).

Arousal is a continuum of sensitivity of the organism to stimuli, both external and internal; facilitates interaction with the environment in a context-specific manner (e.g., under conditions of threat, some stimuli must be ignored while sensitivity to and responses to others is enhanced, as exemplified in the startle reflex), can be evoked by either external/environmental stimuli or internal stimuli (e.g., emotions and cognition); can be modulated by the physical characteristics and motivational significance of stimuli, varies along a continuum that can be quantified in any behavioral state, including wakefulness and low-arousal states including sleep, anesthesia, and coma; can be regulated by homeostatic drives (e.g., hunger, sleep, thirst, sex) (RDoC, 2019).

In late-life depression, apathy is accompanied by functional alterations characterized by low resting-state connectivity of the nucleus accumbens with the amygdala, globus pallidus, thalamus, caudate, putamen, and increased connectivity with the dorsomedial prefrontal cortex and insula (Alexopoulos et al., 2015; Alexopoulos & Arean, 2014)

Both cognitive-behavioral and ecosystem modification strategies are used to cope with apathy, since in severe expressions of apathy, the surrounding context of the individual plays a critical role to mitigate it. Prompts to trigger the action plans for reward exposure are required, for example, checklists, reminders, tape recorders, electronic instructions to start tasks, family and friends acting as prompts (Alexopoulos et al., 2016, 2017; Alexopoulos & Arean, 2014).

### Cognitive Systems: cognitive control

Finally, emotional dysregulation is considered the behavioral expression of *Cognitive Systems* dysfunction, namely the *Cognitive Control* construct. Cognitive control “modulates the operation of other cognitive and emotional systems, in the service of goal-directed behavior, when prepotent modes of responding are not adequate to meet the demands of the current context; control processes are engaged in the case of novel contexts, where appropriate responses need to be selected from among competing alternatives” (RDoC, 2019).

Emotional dysregulation may result from abnormal patterns of functioning of the ventral-rostral anterior cingulate cortex and medial prefrontal cortical areas responsible for appraisal and regulation of emotional functions, as well as limbic subcortical circuits of emotion processing (Alexopoulos et al., 2015; Alexopoulos & Arean, 2014).

The coping strategies are selected from previous experiences and may include modulation (e.g., overwhelming disappointment and anxiety) and management of emotions (e.g., distraction, imagery, relaxation exercises, deep breathing, meditation). The selected strategy is practiced during the session and then autonomously, to experience the difficulties when pursuing plans for reward exposure.

### Research evidence supporting Engage

The strategy to evaluate Engage targeted the assessment of training time, fidelity to practice sessions, reduction in depression severity, longitudinal maintenance of the gains and remission.

Considering the focus on simplification and applicability, Alexopoulos et al. (2015) examined the acquisition of therapeutic skills to administer Engage and the post-training fidelity to treatment in relation to problem-solving therapy. Both social workers and therapists required a shorter training time to be certified in Engage (6.92 h) than in problem-solving therapy. Post-training fidelity was examined by reviewing audiotapes of 63 Engage and 93 problem-solving therapy sessions. Only 12.7% of the Engage sessions required corrective feedback (vs. 36.6% in problem-solving therapy). From the results, the simplification of the language made the interventions more accessible to clinicians. The authors pointed out that Engage is the first psychotherapy using RDoC concepts as a guide to simplification and such an approach may promote fidelity (Alexopoulos et al., 2015).

The efficacy of Engage was further assessed in 39 depressed older adults over 9 weeks, in comparison with 97 patients treated with the problem-solving therapy (Alexopoulos et al., 2015). This therapy was used as a term of comparison given that Engage uses a simplified problem-solving approach (Alexopoulos & Arean, 2014). There were no demographic or clinically significant differences at baseline between the two groups, namely in depression severity, medical burden, disability, and cognitive impairment. Engage did not differ from problem-solving therapy in reducing depression severity. Remission rates for Engage at 6 and 9 weeks were 18.2% and 41.1%, respectively, compared to 13.7% and 35.0% for problem-solving therapy. In a subsequent study (Alexopoulos et al., 2016), and including a sample size of 48 depressed older adults, depression scores sharply declined by 10 points during the 9 weeks of treatment and increased mildly by 4.8 points during the follow-up phase (36 weeks). Additional interventions for negativity bias, apathy, and emotional dysregulation did not impact the results in both studies (Alexopoulos et al., 2015, 2016). Using self-report as a unit of analysis to measure activation and to capture approach motivation and reward learning, it was found that the number and variety of engaged activities, the ability to remain active in the face of difficulties, the sensitivity to reward, and feelings of accomplishment had a sharp increase during the 9 weeks of Engage treatment and a mild decline during follow-up. Only activation predicted depression severity at the end of each period of assessment. An increase of one standard deviation in activation resulted in a decrease in depression scores.

The main results were considered by the authors as a proof of concept for Engage efficacy in reducing depressive symptoms, promoting remission and the maintenance of gains by using behavioral activation techniques of reward exposure and a simplified, personalized intervention in RDoC positive valence systems that also considers specific differences in response to intervention (Alexopoulos et al., 2015, 2016, 2017; Alexopoulos & Arean, 2014).

## Critical analysis of RDoC applicability to empirically supported psychological interventions

Almost a decade has passed since the NIMH launched the RDoC initiative, and there are still few intervention studies published, with some arguing about the difficulties in integrating the matrix into the current modi operandi of psychological intervention (Appelbaum, 2017).

Even though there are scarce empirical studies, which limits the evaluation of RDoC applicability, in the next sections we attempted to critically discuss the merits and drawbacks of RDoC at the conceptual, methodological, and efficacy measurement levels, based on the reviewed empirically based interventions. TARA and Engage are illustrative and represent an effort to conduct RDoC-inspired interventions in naturalistic settings and may expose some advances and difficulties in implementing RDoC in clinical practice. These first efforts in applying RDoC to psychological intervention may be a helpful source of recommendations for future and improved RDoC-tailored psychological interventions.

### Conceptual level

How to reframe psychological interventions based on the constructs of the RDoC matrix and on its transdiagnostic basic mechanisms, rather than on previous clinical diagnosis, represents an innovative postulate that may foster optimally matched treatments for mental disorders by settling specific profiles for intervention from the differential combination of RDoC constructs as targets (Geraldo et al., 2018).

The selection of RDoC constructs as targets in TARA and Engage were guided by the dimensional character of the RDoC matrix, and in the case of TARA, by a transdiagnostic stance. The attribution of a hierarchical value among the selected constructs to define the priorities for intervention illustrated how tailored and less standardized interventions may be conducted (Alexopoulos & Arean, 2014). The individual variability was particularly taken into account in Engage, since the strategies for intervention were dependent on the distinct profiles of depression representing different RDoC-related dysfunctions. TARA also introduced therapeutic objectives along the therapy sessions based on the cascade of purported causality between associated mechanisms in adolescent depression.

It is important to note that Engage and TARA selected distinct constructs to intervention to the same psychopathological phenomenon (depression), following a developmental sensitive framework: older adults vs. adolescents. Reward learning was the focus of interest of Engage and supported the designed strategies for social engagement in older adults, while TARA extended to other interrelated constructs. In fact, TARA included more constructs than Engage, which forces us to think on the optimal set of constructs to be included in an intervention that intends to be both comprehensive and feasible.

At this point, it is critical to acknowledge that the dimensional constructs and the differential interaction among them do not necessarily increase the complexity of the intervention. The RDoC matrix offers a structured guidance that intends to help clinicians defining targets for intervention in a clearer and more precise fashion, working towards simpler though comprehensive therapies. Indeed, it is advocated that the simplified/structured RDoC approach to intervention is a necessary first step for large-scale use (Alexopoulos & Arean, 2014).

However, operationalizing RDoC principles in naturalistic settings is challenging (Sharp et al., 2016).

The RDoC matrix is lacking important psychological constructs (Mittal & Wakschlag, 2017), which limits its applicability to the study of psychopathology and to the design of psychological interventions. For example, the social processes domain used in TARA lacks the detail and precision of other constructs (e.g., negative valence systems). In fact, a recent systematic review on RDoC published research highlights that social processes is one of the least studied domains (alongside with arousal and regulatory systems) (Carcone & Ruocco, 2017). This is a crucial limitation for clinical research, as social processes are central to understanding how psychopathology develops (e.g., Autism Spectrum Disorder) and many intervention strategies capitalize on these processes, whether by the means of delivery (e.g., face-to-face therapy) or by the inclusion of targets for intervention (e.g., develop social skills, increase social support).

An embedded developmental approach is also missing in the definition of RDoC constructs (Mittal & Wakschlag, 2017). As they are currently defined, many are adult based (or at least the units of analysis focus on this age range) or are presented as varying smoothly throughout development (Mittal & Wakschlag, 2017;Patrick & Hajcak, 2016 ; Peterson, 2015). By disregarding that the constructs may be developmentally discontinuous, considerable challenges are posed for their inclusion in developmental and youth intervention studies (Patrick & Hajcak, 2016; Peterson, 2015).

Considering all the conceptual limitations of the matrix to clinical practice, it is important to bear in mind that the matrix is a work in progress (Clark et al., 2017). This means that constructs—or developmental variations of the constructs—found to be meaningful to understand psychopathology and to improve interventions may be integrated at any time into the matrix. Nevertheless, it may also mean that the data collected from the guidelines of the archived versions of the matrix may be out of date quickly and lose the expected impact for guiding future and improved studies. For instance, the positive valence systems domain used in Engage was reconceptualized, and although it was not the case, this change could have called into question the empirical validity in which the intervention was based on.

Another RDoC feature that may impel psychological researchers and clinicians away from using this framework is its focus on biology. As a first implication, the RDoC constructs become unfamiliar to clinicians and their clinical utility is not obvious. Despite the relevance of some constructs for several disorders, there is no clear connection between psychological symptoms, clinical problems, and the constructs, demanding a deeper specification of clinical problems as targets for RDoC intervention research (Patrick & Hajcak, 2016). Moreover, the RDoC matrix assumes that the different units of analyses are equivalent for each construct, which enables researchers to integrate results from different fields and clinicians to use the accumulated knowledge to devise therapeutic interventions (Marková, 2018). As such, translational research searches for the meaning of a certain process in one level of analysis (e.g., biochemical changes in the brain) and also for the result of such changes on other levels of analysis (e.g., behavior and symptoms). However, there seems to be no one-to-one correspondence between units of analysis within the same construct (Peterson, 2015). As we progress through the different levels of analysis, from more internal (e.g., molecules) to more external (e.g., self-reports), there is no isomorphic transfer of meaning; that is, information is lost and new is added (Marková, 2018).

Finally, when RDoC defines mental disorders from a biological framework focused on the neurodevelopmental aspects of psychopathology, there is a risk to claim that alterations in the brain and biology are causing the altered mental states and behavior in a reductionist and linear way (Marková, 2018). This could lead to an internalized (mis)conception of psychological disorders as well as to therapeutic interventions focused solely on biological and pharmacological targets. This would deny the importance of translating the research to psychological interventions and would leave behind the role of the environment for the development of psychopathology. Therefore, it is important to keep a continuous critical analysis on how multifactorial causality in psychopathology is being operationalized in RDoC research.

Biological research on basic mechanisms of psychopathology may be the anchor of RDoC, but a closer look to the translational endeavor shows that RDoC interventions inspired do not rely on reductionism. As stated in RDoC assumptions, an overemphasis on biology would neglect the evidence for a multicausal nature of mental illness. Thus, it is proposed that psychopathology is best conceptualized as a confluence of multiple factors—from biological, developmental, psychosocial, to environmental—that are indeed integrated in the RDoC matrix (Clark et al., 2017). For example, psychological factors were the focus of the reviewed interventions, but the contextual and social factors were also considered. Engage uses ecosystem modification strategies (e.g., the apathy was reduced by family and friends acting as prompts) and the emotion regulation strategies in TARA are practiced in relevant social interactions. Also, in RDoC agenda it is explained that the definition of the constructs around the identification of neural circuit does not mean a causal relation between them. It arose from the necessity to find a central unit of analysis that could easily aggregate research streaming from biology and neuroscience to psychology (Kozak & Cuthbert, 2016), contributing to the definition of testable and valid psychological constructs, which are relevant to human functioning (Clark et al., 2017).

In this line, RDoC may be grounded on the identification of basic mechanisms to intervention—as TARA and Engage did—but the transcultural and universal aspects of these basic mechanisms are of high importance as well. RDoC provides tools and calls for research in large datasets including distinct groups and minorities to test the transcultural validity of its constructs. The adaptation of interventions for distinct cultural and social contexts may contribute to improving the tailoring aspects of RDoC-inspired empirically supported interventions for psychological disorders.

### Methodological level

An innovation introduced by RDoC at the methodological level concerns the non-conservative inclusion criteria when selecting participants for research and intervention (Van Dam et al., 2017). The reduction in inclusion criteria impels towards a real approximation of the psychopathological phenomenon that cuts across diagnostic boundaries and a real representation of the representative sample. For instance, TARA includes individuals with both symptoms of depression and anxiety; two highly comorbid conditions that are usually approached as distinct (and apparently independent) diagnostics. In turn, the intervention in Engage is disorder-specific and reduces the transdiagnostic applicability of the intervention. By not considering psychiatric comorbidity, no conclusions can be drawn about the suitability of Engage in depressed elders with additional psychiatric diagnoses (Alexopoulos et al., 2015). In a more general note, neither TARA nor Engage follow the full directives of RDoC concerning inclusion criteria to the extent they should have included other diagnoses, subclinical expressions and even healthy participants to address the normal-to-abnormal spectrum continuum.

In the case of Engage, these methodological limitations end in a group comparison analysis that loses its central importance in the RDoC preconized dimensional/continuous/transdiagnostic approach. Nevertheless, the between-group comparison between Engage and problem-solving therapy provides stronger evidence than TARA’s study, which did not have a comparison group. The conclusions on TARA are based on a pilot study including a small and age-restricted sample of 26 adolescents followed for 3 months. The comparison Engage vs. problem-solving therapy was, however, conducted without randomization and, again, in a short period of time (9 weeks). The follow-up for 36 weeks did not include a control group. Thus, the main results should be seen as exploratory, remaining unclear whether symptoms remission is specifically related to the intervention. More efforts should be allocated to retrieve valid conclusions on the longitudinal, dimensional, and continuous analysis preconized by RDoC.

Furthermore, the use of self/hetero-reports and behavioral assessments may be a trend in many psychological studies but offers limited evidence. The RDoC matrix brings up other units of analysis (i.e., molecules, physiology, circuits) that should be included to get more robust indicators of efficacy (Mckay & Tolin, 2017). TARA and Engage did not take advantage of this novel approach to measure efficacy and have not included biological or other units of analysis, although they used neuroscientific evidence to consubstantiate their interventions. Using the RDoC’ units of analysis to search for evidence of validity is a promising new venue, even if including assessment measures from several research fields requires the interplay between areas of knowledge, multidisciplinary teams, and longer assessment procedures, making this a costly and demanding endeavor (Geraldo et al., 2018). As such, it is not clear how it will be achieved in mainstream intervention studies.

### Efficacy measurement level

Until now, most evidence-based interventions in psychology have been based on repetitive demonstrations of efficacy (Clark et al., 2017; David & Montgomery, 2011). This means that the efficacy of the psychological interventions has been tested in a vacuum regarding the validity of the psychological theories that gave rise to these same interventions.

Since the RDoC matrix is empirically driven, a major advantage is that it serves as a tool to inform about psychological theories. Moreover, researchers are now able to collect evidence for the efficacy of the intervention by using measures from the different levels of analysis described in the selected constructs for intervention. That is, RDoC not only searches for empirically based theories about psychological mechanisms of psychopathology but also provides units of analysis that allows to measure the efficacy of the intervention. This fosters empirically supported interventions in a double sense. From the RDoC matrix, TARA, and Engage searched for validity evidence on the psychopathological theories to define the constructs of interest, change mechanisms, and consequent intervention strategies, as well as to measure the impact of the intervention by using some of the proposed units of analysis.

Nevertheless, some shortcomings may be exposed when considering the RDoC measures included in each unit of analysis and how they relate to each other.

Efforts undertaken to establish psychometric properties of the psychophysiological measures are scarce in contrast with self-reports. The convergent and divergent validity of the psychophysiological measures, as well as their reliability, need to be empirically established (Patrick & Hajcak, 2016; Peterson, 2015). This is a clear obstacle to asserting that these measures are valid indices of the psychological constructs.

Patrick and Hajcak (2016) further call our attention to the difficulties in achieving high or even moderate correlations between different units of analysis included in the same construct, due to the domain-specific/method variance. This taps on our previous discussion regarding the lack of a precise, one-to-one, correspondence between units of analysis within the same construct (Marková, 2018); that is, information about the construct is not exactly the same when moving along the units of analysis. Even so, the domain-specific/method variance problem is not specific of the RDoC and is expected to be found in multi-level assessments. Investigators (e.g., Yancey, Venables, & Patrick, 2016) have pointed methodological approaches to address this issue. The computation of cross-domain composites using structural equation modeling and multidimensional item-response modeling may be sound alternatives, but demand for large datasets that are required to ensure high statistical power when testing all the hypotheses paths. For instance, the analysis of the interaction between risk factors, moderation, and mediation effects is complex and demanding but is necessary to provide a comprehensive picture of RDoC postulates. From the provided (complex) picture, it remains difficult to accommodate mediation and moderation effects in the analysis of efficacy as measured from different units of analysis, while dissociating these effects from risk factors.

There were some efforts of TARA and Engage in this direction when considering the hierarchical organization of the constructs for intervention and the sample characteristics. Engage clearly dissociates the main construct of interest as the risk factor (i.e., reward learning) from other constructs that may mediate and potentiate the results (i.e., loss, arousal and cognitive control constructs as reducing, respectively, the negativity bias, apathy, and emotional dysregulation). TARA further tested a larger set of symptoms to prove the transdiagnostic applicability of the intervention while prioritizing constructs that purportedly have a greater impact on the development of depression and anxiety in adolescents (e.g., Sustained Threat) vs. other age groups. Considering that different mechanisms are implicated in different interventions, the inclusion of mediation and moderation effects may increase our understanding for whom the intervention works and improve the development of synergistic treatments, contemplating the maximization of active therapeutic components and the minimization of antagonistic ingredients (Hershenberg & Goldfried, 2015)

Thus, and despite the methodological limitations previously presented, there are some evidence for the efficacy of TARA in what regards the mechanisms of change. The inclusion of the purported psychopathological mechanism—RDoC construct (e.g., sustained threat—amygdala hyperreactivity), and intervention strategies targeting this construct (e.g., increase vagal efference to lower amygdala hyperreactivity through deep breathing) was related with the expected outcomes, both at process (e.g., better regulatory skills and sleep) and symptom levels (e.g., depression). These findings may indicate that the targeted constructs are relevant for empirically supported interventions as they were associated with critical mechanisms of change in the psychopathological phenomenon (McKay & Tolin, 2017). Contrarily, Engage was focused uniquely on symptom remission as measured by the included self-reports. Self-report measures focused on symptomology are not foreseen in the RDoC matrix to measure the change on criteria for clinical diagnosis, but rather to assess the change on mechanisms that have a close link with the maladaptive expression of the construct. This limits the analysis of efficacy under a RDoC approach and is associated with the traditional way of conceiving psychopathology. The use of self-reported measures described in the RDoC matrix should be included to test the convergent validity of the constructs and to measure the efficacy in a consistent way with the RDoC proposals of accumulating knowledge in a large (international) dataset. Nonetheless, the RDoC matrix is not intended to be prescriptive, and it may be important to maintain the link between RDoC and symptomatology to keep the results comparable and interpretable, at least at an early stage of research.

In fact, the few and limited evidence to date, alongside with the differences in the methodological and efficacy measurement levels between RDoC and more traditional approaches, may make it difficult to assess if RDoC interventions are better than the gold standard ones (e.g., cognitive-behavioral therapy). Despite the focus of RDoC on continuous and longitudinal data instead of taxonomic analyses, the gap between the two approaches may be bridged, in a first moment, by conducting studies with similar designs (e.g., randomized control trials) (David & Montgomery, 2011) or by fostering comprehensive analytical procedures (e.g., regression-based models).

## Conclusion

Presently and taking into consideration that there is limited research on RDoC-inspired psychological interventions, the depth and practicality of these applications remains largely hypothetical. The paucity of published RDoC-inspired psychological interventions may relate to the limitations and misconceptions described above at a conceptual, methodological and efficacy measurement levels. The small number of published interventions precludes the ambition of collecting large datasets in real-life intervention contexts and of including representative psychiatric populations with complex and multifaceted symptom profiles (Alexopoulos et al., 2015; Hershenberg & Goldfried, 2015), that would lead to a real assessment on RDoC applicability.

Nevertheless, RDoC has several contributes to advance our knowledge on psychopathology and holds promise to be a profitable framework for psychological researchers and clinicians. It may inspire more valid understanding of psychological functioning and psychopathology and in turn influence the development of better evidence-based psychological interventions. In this review, we tried to highlight the potentials of the RDoC initiative and explore the main criticisms and expected limitations of its directives in real-life intervention studies, hoping that it will sparkle new attempts in applying RDoC to psychological intervention.

## Data Availability

Not applicable

## Abbreviations

DSM: Diagnostic Manual of Mental Disorders
RDoC: Research Domain Criteria
TARA: Training for awareness resilience and action

## References

[CR1] Alexander, G. M., Huang, Y. Z., Soderblom, E. J., He, X. P., Moseley, M. A., & McNamara, J. O. (2017). Vagal nerve stimulation modifies neuronal activity and the proteome of excitatory synapses of amygdala/piriform cortex. Journal of neurochemistry, 140(4), 629-644. 10.1111/jnc.13931 PMC6537100 27973753

[CR2] Alexopoulos GS, Arean P (2014). A model for streamlining psychotherapy in the RDoC era: The example of “Engage”. Molecular Psychiatry.

[CR3] Alexopoulos GS, O’Neil R, Banerjee S, Raue PJ, Victoria LW, Bress JN (2017). “Engage” therapy: Prediction of change of late-life major depression. Journal of Affective Disorders.

[CR4] Alexopoulos GS, Raue PJ, Gunning F, Kiosses DN, Kanellopoulos D, Pollari C (2016). Engage therapy: Behavioral activation and improvement of late-life major depression. American Journal of Geriatric Psychiatry.

[CR5] Alexopoulos GS, Raue PJ, Kiosses DN, Seirup JK, Banerjee S, Arean PA (2015). Comparing engage with PST in late-life major depression: A preliminary report. American Journal of Geriatric Psychiatry.

[CR6] Appelbaum Paul S. (2017). Moving Toward the Future in the Diagnosis of Mental Disorders. Psychological Science in the Public Interest.

[CR7] Beck AT, Bredemeier K (2016). A unified model of depression. Clinical Psychological Science.

[CR8] Berkovich-Ohana A, Glicksohn J, Goldstein A (2012). Mindfulness-induced changes in gamma band activity – implications for the default mode network, self-reference and attention. Clinical Neurophysiology.

[CR9] Blom EH, Duncan LG, Ho TC, Connolly CG, LeWinn KZ, Chesney M (2014). The development of an RDoC-based treatment program for adolescent depression:“Training for Awareness, Resilience, and Action”(TARA). Frontiers in Human Neuroscience.

[CR10] Blom, E. H., Tymofiyeva, O., Chesney, M. A., Ho, T. C., Moran, P., Connolly, C. G., … Yang, T. T. (2017). Feasibility and preliminary efficacy of a novel RDoC-based treatment program for adolescent depression: “Training for Awareness Resilience and Action” (TARA)—A Pilot Study. *Frontiers in Psychiatry*, *7*. 10.3389/fpsyt.2016.00208. 10.3389/fpsyt.2016.00208 PMC5237634 28138319

[CR11] Carcone, D., & Ruocco, A. C. (2017). Six years of research on the National Institute of Mental Health’s Research Domain Criteria (RDoC) Initiative: A systematic review. *Frontiers in Cellular Neuroscience*, *11*. 10.3389/fncel.2017.00046. 10.3389/fncel.2017.00046 PMC5334510 28316565

[CR12] Clark D, Konduru N, Kemp A, Bray S, Brown E, Goodyear B, Ramasubbu R (2018). The impact of age of onset on amygdala intrinsic connectivity in major depression. Neuropsychiatric Disease and Treatment.

[CR13] Clark LA, Cuthbert B, Lewis-Fernández R, Narrow WE, Reed GM (2017). Three Approaches to understanding and classifying mental disorder: ICD-11, DSM-5 , and the National Institute of Mental Health’s Research Domain Criteria (RDoC). Psychological Science in the Public Interest.

[CR14] Clementz BA, Sweeney JA, Hamm JP, Ivleva EI, Ethridge LE, Pearlson GD (2016). Identification of distinct psychosis biotypes using brain-based biomarkers. American Journal of Psychiatry.

[CR15] Cullen KR, Westlund MK, Klimes-Dougan B, Mueller BA, Houri A, Eberly LE, Lim KO (2014). Abnormal amygdala resting-state functional connectivity in adolescent depression. JAMA Psychiatry.

[CR16] Cuthbert, B. N., & Insel, T. R. (2013). Toward the future of psychiatric diagnosis: The seven pillars of RDoC. *BMC Medicine*, *11*(1). 10.1186/1741-7015-11-126. 10.1186/1741-7015-11-126 PMC3653747 23672542

[CR17] Cuthbert BN, Kozak MJ (2013). Constructing constructs for psychopathology: The NIMH Research Domain Criteria. Journal of Abnormal Psychology.

[CR18] David D, Montgomery GH (2011). The scientific status of psychotherapies: A new evaluative framework for evidence-based psychosocial interventions. Clinical Psychology: Science and Practice.

[CR19] Drysdale AT, Grosenick L, Downar J, Dunlop K, Mansouri F, Meng Y (2016). Resting-state connectivity biomarkers define neurophysiological subtypes of depression. Nature Medicine.

[CR20] Emmelkamp PMG, David D, Beckers T, Muris P, Cuijpers P, Lutz W (2014). Advancing psychotherapy and evidence-based psychological interventions. International Journal of Methods in Psychiatric Research.

[CR21] Ferster CB (1973). A functional analysis of depression. American Psychologist.

[CR22] Fowler CH, Miernicki ME, Rudolph KD, Telzer EH (2017). Disrupted amygdala-prefrontal connectivity during emotion regulation links stress-reactive rumination and adolescent depressive symptoms. Developmental Cognitive Neuroscience.

[CR23] Geraldo Andreia, Azeredo Andreia, Pasion Rita, Dores Artemisa Rocha, Barbosa Fernando (2018). Fostering advances to neuropsychological assessment based on the Research Domain Criteria: The bridge between cognitive functioning and physiology. The Clinical Neuropsychologist.

[CR24] Hamilton JP, Furman DJ, Chang C, Thomason ME, Dennis E, Gotlib IH (2011). Default-mode and task-positive network activity in major depressive disorder: Implications for adaptive and maladaptive rumination. Biological Psychiatry.

[CR25] Hershenberg R, Goldfried MR (2015). Implications of RDoC for the research and practice of psychotherapy. Behavior Therapy.

[CR26] Insel T, Cuthbert B, Garvey M, Heinssen R, Pine DS, Quinn K (2010). Research Domain Criteria (RDoC): Toward a new classification framework for research on mental disorders. American Journal of Psychiatry.

[CR27] Kerestes R, Harrison BJ, Dandash O, Stephanou K, Whittle S, Pujol J, Davey CG (2015). Specific functional connectivity alterations of the dorsal striatum in young people with depression. NeuroImage: Clinical.

[CR28] Koszycki D, Thake J, Mavounza C, Daoust J-P, Taljaard M, Bradwejn J (2016). Preliminary investigation of a mindfulness-based intervention for social anxiety disorder that integrates compassion meditation and mindful exposure. The Journal of Alternative and Complementary Medicine.

[CR29] Kozak MJ, Cuthbert BN (2016). The NIMH Research Domain Criteria Initiative: background, issues, and pragmatics. Psychophysiology.

[CR30] Levant RF (2005). *Report of the 2005 presidential task force on evidence-based practice*.

[CR31] Marchand William R. (2012). Self-Referential Thinking, Suicide, and Function of the Cortical Midline Structures and Striatum in Mood Disorders: Possible Implications for Treatment Studies of Mindfulness-Based Interventions for Bipolar Depression. Depression Research and Treatment.

[CR32] Marková IS (2018). Translational neuroscience and psychiatry: A conceptual analysis. Journal of Evaluation in Clinical Practice.

[CR33] McKay Dean, Tolin David F. (2017). Empirically supported psychological treatments and the Research Domain Criteria (RDoC). Journal of Affective Disorders.

[CR34] Mittal VA, Wakschlag LS (2017). Research Domain Criteria (RDoC) grows up: Strengthening neurodevelopment investigation within the RDoC framework. Journal of Affective Disorders.

[CR35] Morris SE, Rumsey JM, Cuthbert BN (2014). Rethinking mental disorders: The role of learning and brain plasticity. Restorative Neurology and Neuroscience.

[CR36] Pasion R, Paiva TO, Fernandes C, Almeida R, Barbosa F (2018). ERN modulation under sustained threat: A pre-registered report. Journal of Research in Personality.

[CR37] Patrick CJ, Hajcak G (2016). RDoC: Translating promise into progress. Psychophysiology.

[CR38] Peterson BS (2015). Editorial: Research Domain Criteria (RDoC): a new psychiatric nosology whose time has not yet come. Journal of Child Psychology and Psychiatry.

[CR39] Price JL, Drevets WC (2012). Neural circuits underlying the pathophysiology of mood disorders. Trends in Cognitive Sciences.

[CR40] RDoC. (2018). RDoC matrix - version 4. Retrieved from https://www.nimh.nih.gov/research/research-funded-by-nimh/rdoc/constructs/rdoc-snapshot-version-4-saved-5-30-18.shtml

[CR41] RDoC. (2019). RDoC constructs. Retrieved from https://www.nimh.nih.gov/research-priorities/rdoc/constructs/index.shtml

[CR42] Roberts MC, Blossom JB, Evans SC, Amaro CM, Kanine RM (2017). Advancing the scientific foundation for evidence-based practice in clinical child and adolescent psychology. Journal of Clinical Child & Adolescent Psychology.

[CR43] Sharp C, Christopher Fowler J, Salas R, Nielsen D, Allen J, Oldham J (2016). Operationalizing NIMH Research Domain Criteria (RDoC) in naturalistic clinical settings. Bulletin of the Menninger Clinic.

[CR44] Shoham V, Rohrbaugh MJ, Onken LS, Cuthbert BN, Beveridge RM, Fowles TR (2014). Redefining clinical science training: Purpose and products of the delaware project. Clinical Psychological Science.

[CR45] Taren AA, Gianaros PJ, Greco CM, Lindsay EK, Fairgrieve A, Brown KW (2015). Mindfulness meditation training alters stress-related amygdala resting state functional connectivity: A randomized controlled trial. Social Cognitive and Affective Neuroscience.

[CR46] Van Dam NT, O’Connor D, Marcelle ET, Ho EJ, Cameron Craddock R, Tobe RH (2017). Data-driven phenotypic categorization for neurobiological analyses: Beyond DSM-5 labels. Biological Psychiatry.

[CR47] Yancey JR, Venables NC, Patrick CJ (2016). Psychoneurometric operationalization of threat sensitivity: Relations with clinical symptom and physiological response criteria. Psychophysiology.

